# A generalized model for communicating individuality through teleost swim bladder modulation

**DOI:** 10.1242/bio.023515

**Published:** 2018-04-30

**Authors:** Cameron A. Matthews, Pierre-Philippe J. Beaujean

**Affiliations:** 1 Naval Surface Warfare Center, Panama City Division, 110 Vernon Ave, Panama City, FL 32407 USA; 2 Florida Atlantic University, Department of Ocean and Mechanical Engineering, 101 North Beach Road, Dania Beach, FL 33004, USA

**Keywords:** Biomodulation, Bioresonance, Bioacoustics

## Abstract

Arguments have been made for and against the traditional swim bladder model as a primary component of fish vocalization. This paper presents arguments for decoupled forced and resonant responses being extractable features within a variable air volume. As such, a mechanical analog is used to show how envelope modulation may be used by some species to identify air volume and consequently size in conspecifics. These arguments consider how an arbitrary fish may apply a genetic strategy of forcing vocalization through slow, fast, or both slow and fast sonic musculature while amplitude modulating via swim bladder. The classic resonant bubble model is revised to account for a hypothetical carrier signal resonance associated with static or varying volume. In the absence of live specimens, a test is conducted in different cylindrical structures with equally sized air volumes. First, a proposed method for extraction of swim bladder volume features through blind amplitude demodulated signals in the time and frequency domain is applied. Second, a proposed method for extraction of swim bladder volume features through cyclostationary analysis of the cross-spectral coherent spectra of the modulated and demodulated signal is applied. Both methods take average frequency content as derived by the prescribed signal processing techniques as the input to the correlator functions used to identify air volumes. Vocalizations of *Epinephelus guttatus*, or more commonly known as the red hind grouper, are used as test signals.

## Abstract (precis)

**Summary:** This paper considers how fish may have evolved methods to utilize modulation of their bladders to convey additional information.

## INTRODUCTION

The swim bladder model for resonance as defined in Ladich (2015) is understood to be one of the most widely accepted approaches to characterizing vocalization from fish who apply muscle contractions across the swim bladder to generate sound. Normal mode analysis can yield varied outputs for any particular geometry being actuated by a source (Frisk, 1994). Evidence has been found to support that some fish are at least sensitive to the effects of envelope modulation (McKibben and Bass, 2001). Researchers are also presenting challenges to the conventional resonating bubble model (Fine, 2012) in terms of understanding how fish apply its use in vocalizing. When considering the possibility of a time and envelope modulation dependent strategy in terms of the findings in McKibben and Bass (2001), it becomes of interest to consider possible biological rationale for developing such proclivities.

## RESULTS

Observations were made on a controlled dataset, where multiple records were collected using 4 inch long standard PVC pipe sections of 1.5, 2, 3 and 4 inch diameter.

## DISCUSSION

This paper presents a methodology for isolating envelope modulation characteristics associated with resonance of air cavities in water being impulsively excited by an external source. As a means of controlling the signal output, an identical test reference vocalization with no variation in power was played through four varying air volumes, and each air volume was correctly assessed by both methods considered. The intent of showing two methods is to corroborate each other. Further, the air volumes were static, implying the most consistent possible modulation scheme as described in Eqn 2 with respect to radius of the air volume. It is important to consider some of the assumptions in developing a successful strategy for accurately estimating the air volumes used as test targets.

With respect to static power, this was intentional with respect to the candidate fish species selected for vocalization; no references on signal power (*IV*) characteristics of the red hind were discovered in the literature review. Relatives of the red hind have been analyzed from a transmission power standpoint (Hazlett and Winn, 1962), but not with enough detail to differentiate size between referenced power sources. With respect to the pivotal behaviors typically seen as associated with vocalization, specifically territorial assertion and mating ritual, Some fish have indeed exhibited correlations in mass and swim bladder size (Fine et al., 2001; Suthers et al., 2016; Ali et al., 2016). If the frequency is indeed coupled with the radius of the swim bladder, then the frequency drop indicated in Suthers et al. (2016) could potentially be indicative of an attempt to maintain a particular modulation effect about an increasing swim bladder volume.

Multipath effects of the pipes were completely neglected in consideration. The pipes may be expected to act as nearly perfect Von Neumann boundaries for the frequencies of interest and have relatively long periods of bounce along the pipe walls affecting linear summing at the receiver. This does not preclude multipath interference from contributing to variations in spectral profile, though none were visibly observed in the collected records.

The resonant bubble model does not strictly require a spherical reverberation, though in the classical form in Ladich (2015) it does not consider the complete effects of normal modes transferring in the wall as lobed or otherwise geometrically defined waves. All of these effects, should they be present, could contribute to variations in the signal envelope in either time or time-frequency analysis. The air bubble effect of a rubber balloon in a pipe does not provide the same biological controls as a fish in terms of damping, movement, etc.

## MATERIALS AND METHODS

To analyze the effect of temporally varying modulation, we first consider the basic problem to be one of impulse (generated in the musculature of the fish) and impulse response (generated in the swim bladder). A classical analysis tool can be found in convolution for separation of such a signal using the definitions found in Ifeachor and Jervis (2002):
(1)
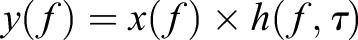
In (1), an impulse signal is presumed to be convolved in the time domain by an impulse response signal and thus multiplied in the frequency domain. This simple model helps describe the effects seen in McKibben and Bass (2001). An altered model can be generated by introducing an ideal filter model as described in Bianchi and Sorrentino (2007). This model implies that the output signal will have a form of carrier signal or a ‘carrier ID’ for a receiver set capable of demodulating both the carrier impulse response, the information signal, and the carrier ID signal. The results in McKibben and Bass (2001) show the capability of a fish being capable of recognizing modulation effects, and at least suggest the possibility of a listening fish being able to infer information from such a modulation such as bladder size and in turn the size of the vocalizing fish.

**Fig. 1.**
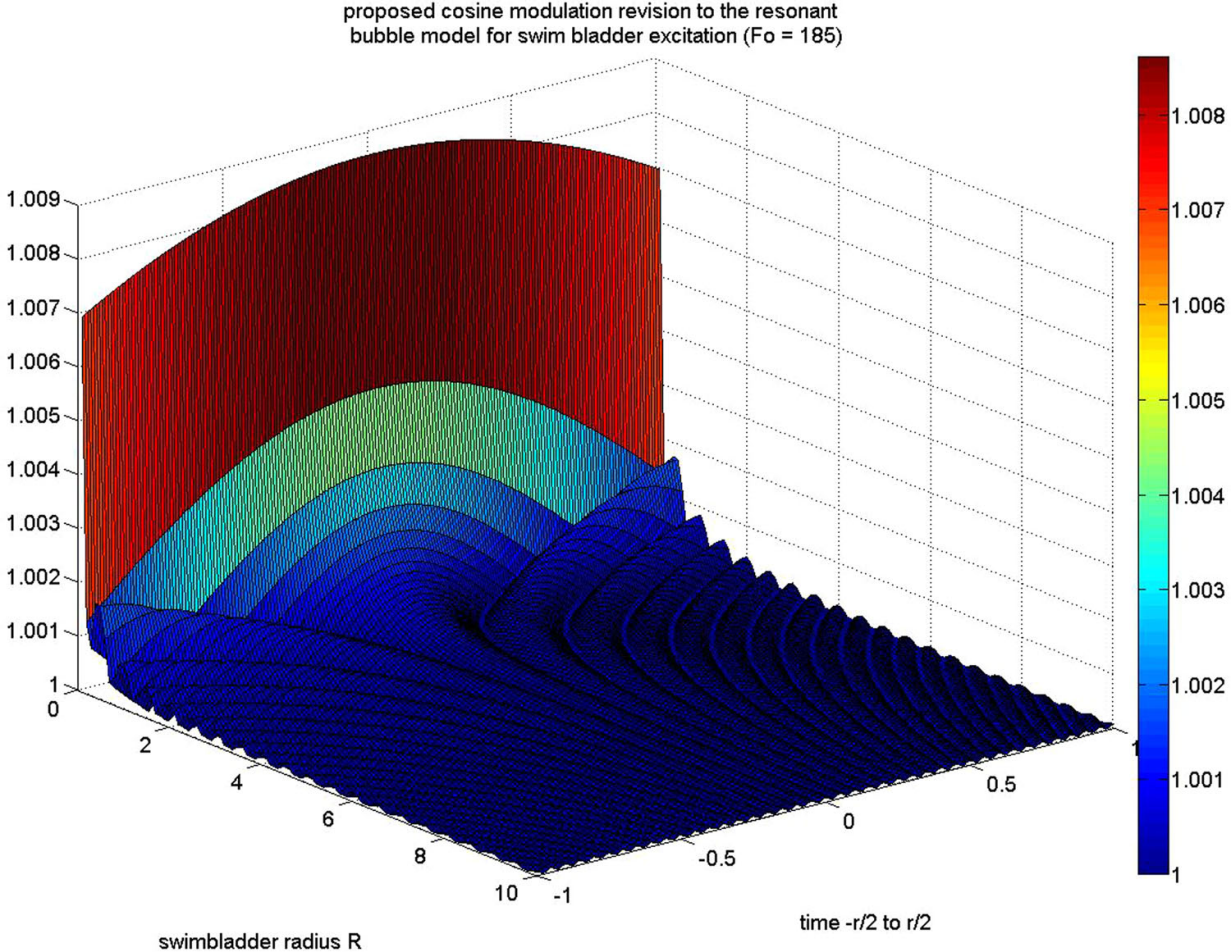
**Raised cosine example model** **of decay in peak modulation amplitude with increasing bladder radius side-by-side with decay in decoupled frequency increase and modulation drop-off.** For a specific frequency F=Fo=185 Hz. Note increase in ripple effect as radius increases in cm while peak modulation drives to unity. Environmental factors are set static for fresh water at a depth of ≈0.1 m.

Based on this possibility, a model is first constructed about the conventional resonant bubble model described in Ladich (2015) for estimating frequency as a function of swim bladder radius and environmental factors. As a variation to this model, we consider an impulse and impulse response model, where the musculature acts as an impulsive force while the bladder acts as an impulse response. For the purposes of this paper, which are to isolate a general estimate of the impulse response, the model is not concerned with features associated with the forcing function of the musculature. The model incorporates time as a variable to indicate the effects as a function of the period of the vocalization. The vocalization over time *t* is defined over a vocalizing record length *r*, so that *t* varies between - *r*/2 and *r*/2. The vocalization is centered at an epoch of time *t*=0:
(2)
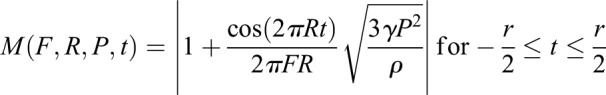
As shown in Fig. 1, the variable *R* represents the radial distance in cm, the ratio of specific heats is *γ*=1.4, *P* is the combined atmospheric and hydrostratic pressure in Pascals (Pa) and *ρ* for the mass density of water in atmosphere. Specific heat, hydrostatic pressure and water mass density remain unchanged in this model, with forcing function as related to the musculature having a frequency *F*. This revision to the model as described in Ladich (2015) describes a generalized modulation signal *M*(*t*) coupled to an impulsive information signal *x*(*t*) (the impulse or comb output of the fish musculature) and the impulse response signal *y*(*t*) (the bladder impulse response).

**Fig. 2.**
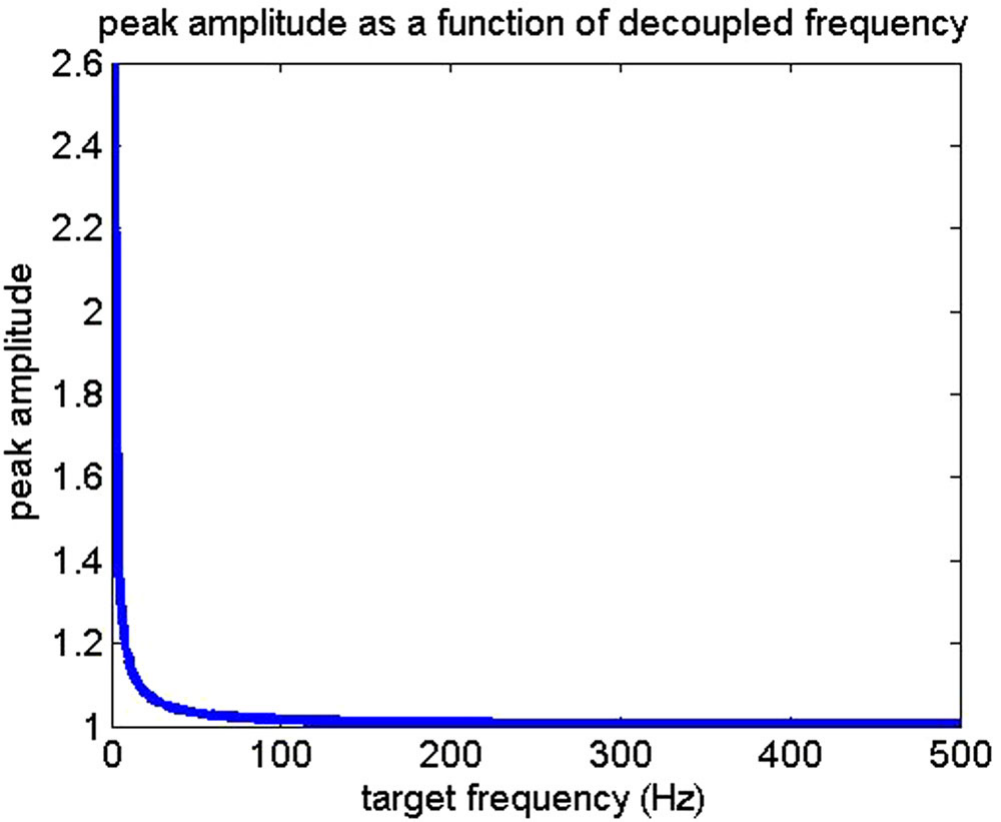
**Example of decay in peak modulation amplitude as a function of decoupled frequency over the previous range of radii and unit time.** Environmental factors are assumed constant for fresh water at a depth of ≈0.1 m.

The implication of the absolute value for *M*(*t*) indicates the form of a raised cosine modulation as the format for the idealized filter. Presuming on a modulation/demodulation strategy in a given species, McKibben and Bass (2001) provides the basis for the presence of an identifying feature used by gravid female midshipmen to identify high value mates – the filter model idealizes with age as a function of the males' swim bladder. The model also presents the possibility that a swim bladder can be used to infer mass of an individual conspecific. If the characteristic modulation can be used to identify a range of bladder volumes associated with mass, then identifying traits of the vocalizing fish species associated with various masses may be extractable features to the observing conspecific. The consequence of this modulation leads to potential value in vocalizing at low frequencies, even in environments inhospitable to low frequency acoustic transmission, as described in Fig. 2.

With regard to a static tone, as radius increases in a swim bladder, the proposed model function takes on a decaying epoch toward unity with an increase in ripple frequency but a decrease in ripple amplitude. An implication of this model is that the peak modulation amplitude drops off significantly as frequency *F* of the forcing function increases. The next pair of figures illustrates these decay models over a single period of unit vocalization time for a hypothetical raised cosine distribution strategy, where a magnitude operator is applied to (1) for a radial variation of 1–10 cm.

Within the model described, it is possible to estimate any number of resonant modulation strategies on the part of the fish, including variable compression of the air bladder to vary the output of *M*(*t*). The swim bladder modulation effect is essentially an all-pass filter for the vocalization, with passband ripple and natural curvature over the ideal 1-D filter envelope [it is important to note that M(t) is a 1-D function, the previous figure would a line drawn through it somewhere to define M(t)]. As the frequency increases, peak cyclical effects associated with the swim bladder approach unity, indicating that the function described in (2) will be driven to unity – essentially an ideal all-pass filter. This supports the evolutionary strategies described in Ladich (2015) regarding low frequency acoustic communication and shallow running teleost fishes if swim bladder modulation features do in fact help identify attractive mates.

The proposed modulation is expected to be, relatively speaking, highly overshadowed by the information signal. As such, in the following section, we consider a well-defined and strong red hind grouper vocalization played through several increasing diameters of pipe filled with a roughly spherical and equally increasing air volume in a typical rubber balloon. It should be noted that any acoustic signal could be applied as the input to the model; the intent is to measure effects of an increasing air volume on the sound passing through it.

**Fig. 3.**
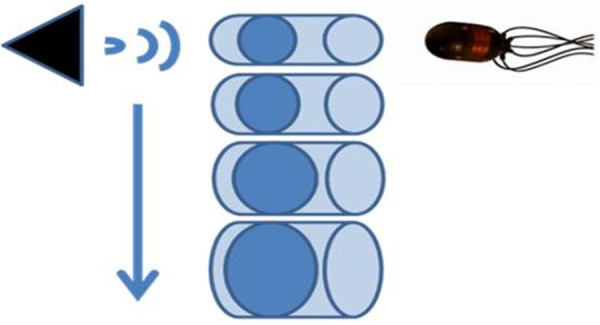
**Source, test air volumes and receiver setup.** A speaker plays a red hind grouper vocalization at fixed power underwater at approximately 1 m of depth at the surface of the air volume while a Wilcoxon 205 model acoustic vector sensor records on the pressure channel. The test is repeated for increasing air volumes of 1.5, 2, 3 and 4 inches (3, 4, 6, 8 cm) respectively of pipe and air diameter.

In order to measure effects in a controlled setting, a physical analog was constructed to allow variations in the air volume for a static test audio file to be played through. Fig. 3 shows a diagram of the test set-up.

**Fig. 4.**
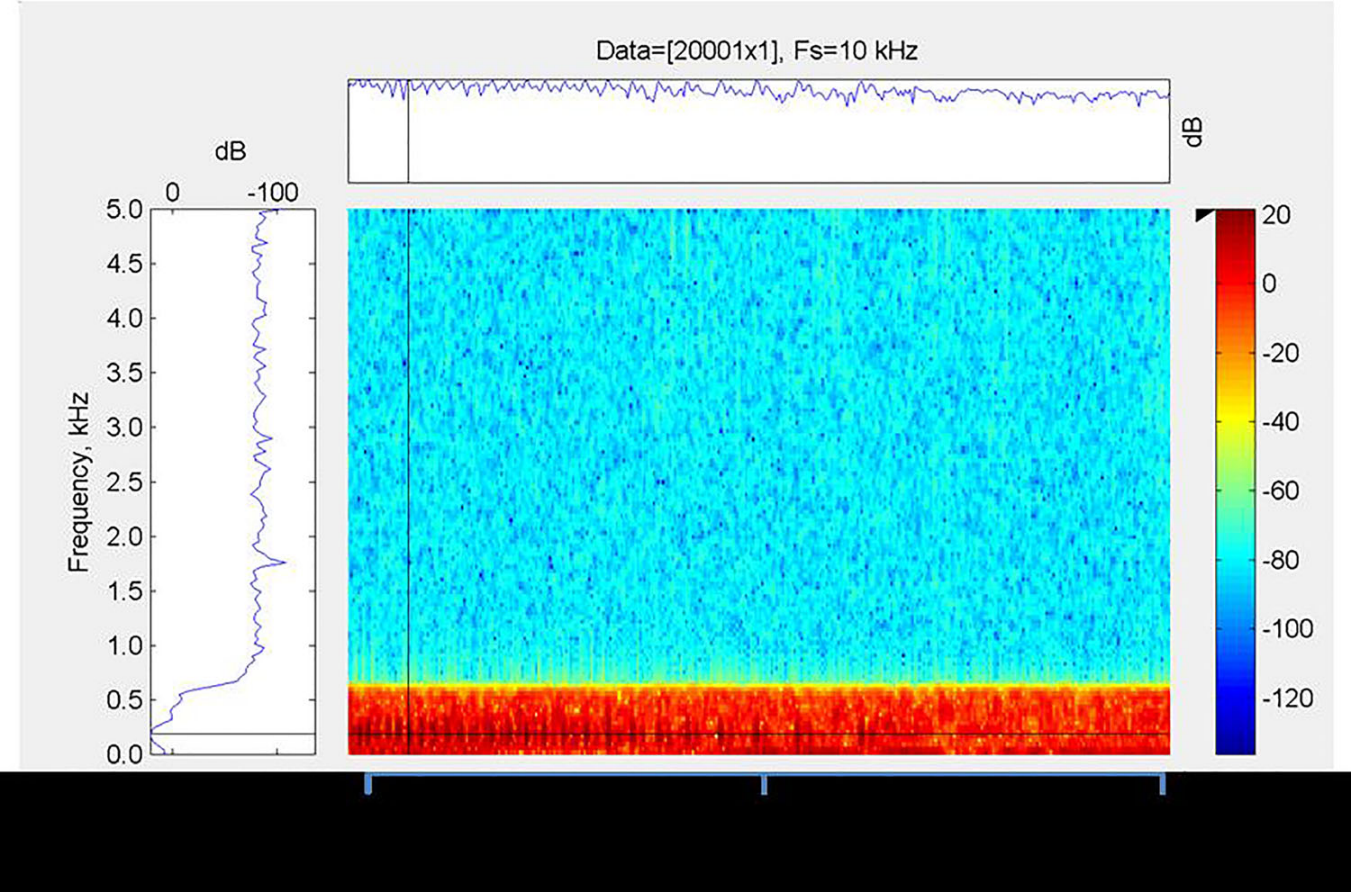
**Spectral content of the test signal applied to varying air volumes.** Note that the signal is exactly 2 s in duration, sampled at 10 kHz. Data provided by Florida Atlantic University (FAU) are low-pass filtered and shown in a spectrogram, with crosshair demarcation lines showing fundamental 185 Hz tone peak and envelope of growl from a red hind grouper. All values shown are dB re 1µPa. The representation is generated with the Mathworks Matlab R2012b product.

With a general framework for analysis and an absence of live specimens to work with, we consider the model thus far in terms of a static volume with fish vocalizations played back through the static volume increased sequentially. The original file is shown below in Short Time Fourier Transform (STFT) – often referred to as spectrogram – format. The reference signals' spectral content is shown in Fig. 4.

In analyzing the signal, methods as described in work by E. C. Like (Non-Cooperative Modulation Recognition Via Exploitation of Cyclic Statistics, MSc Thesis, Wright State University, 2007), A. F. Jr. Lima Analysis Of Low Probability Of Intercept (Lpi) Radar Signals Using Cyclostationary Processing, MSc Thesis, Naval Post-Graduate School, 2002] and Antoni (2007) are applied as well as a general twist on classical demodulation schemes. In terms of general STFT analysis, the demodulation of the signal across the time–frequency domain is expected to yield the base information of the fish vocalization. If the modulation characteristics are consistent with a modulo-resonance model such as the one put forth, a separable impulse response should remain after removing information signal.

**Fig. 5.**
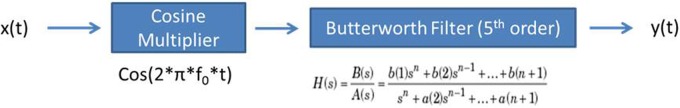
**Flow diagram of the demodulation process as described in Mathworks (2016).**

As an assumption in developing the demodulation strategy, the same methods in McKibben and Bass (2001) are presumed upon regarding amplitude modulation. The blind demodulation estimate also presumes on the fish applying Single Side Band (SSB) amplitude demodulation. The Amplitude Modulation SSB (AM-SSB) model as shown in Fig. 5 is attractive not only as a possible physiological effect of the damping swim bladder wall but also as a carrier suppression tool. The intent of this operation is to decorrelate impulse and impulse response to extract a hypothetical carrier identification signal. The demodulation routine then becomes the signal multiplied by a cosine function using the fundamental of the presumed vocalization [this is readily achieved for red hind vocalization – potentially any communication signal – with peak-finding in energy detectors following a front-end speech detection process, see Matthews and Beaujean (Edge Detection of Red Hind Grouper Vocalizations in the Littorals, Society for Photonics and Imaging Electronics, Buried and Obscured Objects Detection Session, Defense Security Systems, 2016) and C. A. Matthews (Acoustic Tonal And Vector Properties Of Red Hind Grouper Vocalizations, Doctoral Thesis, Florida Atlantic University, 2017)]. The following flow diagram describes the process in detail, which consists in low-pass filtering the modulated signal through a 5th order Butterworth filter as described in Bianchi and Sorrentino (2007).

When the input signal *x* is demodulated, it yields the information signal, termed *y*. When these signals are decorrelated, the remainder is expected to yield carrier signal information, which can then be cross-correlated as a template to an input signal *z*. First, we consider an arbitrary overlap and frequency precision for conversion to time–frequency, where all frequencies *k* being considered, an approximation of the frequency content over time *t* is estimated for *x,y* and *z*, respectively.
(3)
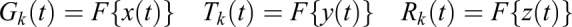
The expectation, or mean, denoted by *E*{}, is applied for the deconvolved frequency content over time for Regions of Interest (ROI) that are germane to the feature set as a means of smoothing information content in frequency *f* about a region of frequencies *k*(*m:n*) of interest contained within *f*. In the case of the red hind grouper for example, the feature set is expected to be contained within the region about 185 Hz as previously considered, such that 150≤*f*≤200 Hz. These signals are then integrated for lag τ.
(4)
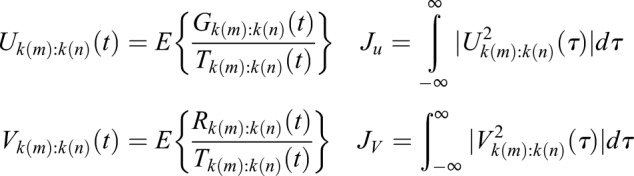
The resulting normalized cross correlation is then derived and labelled *Z_1_*:
(5)
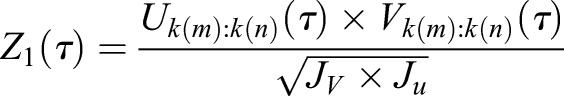
As noted, average frequency content is used since the true modulating signal is unknown, but presumed to reside within the available communication band of the information signal.

**Fig. 6.**
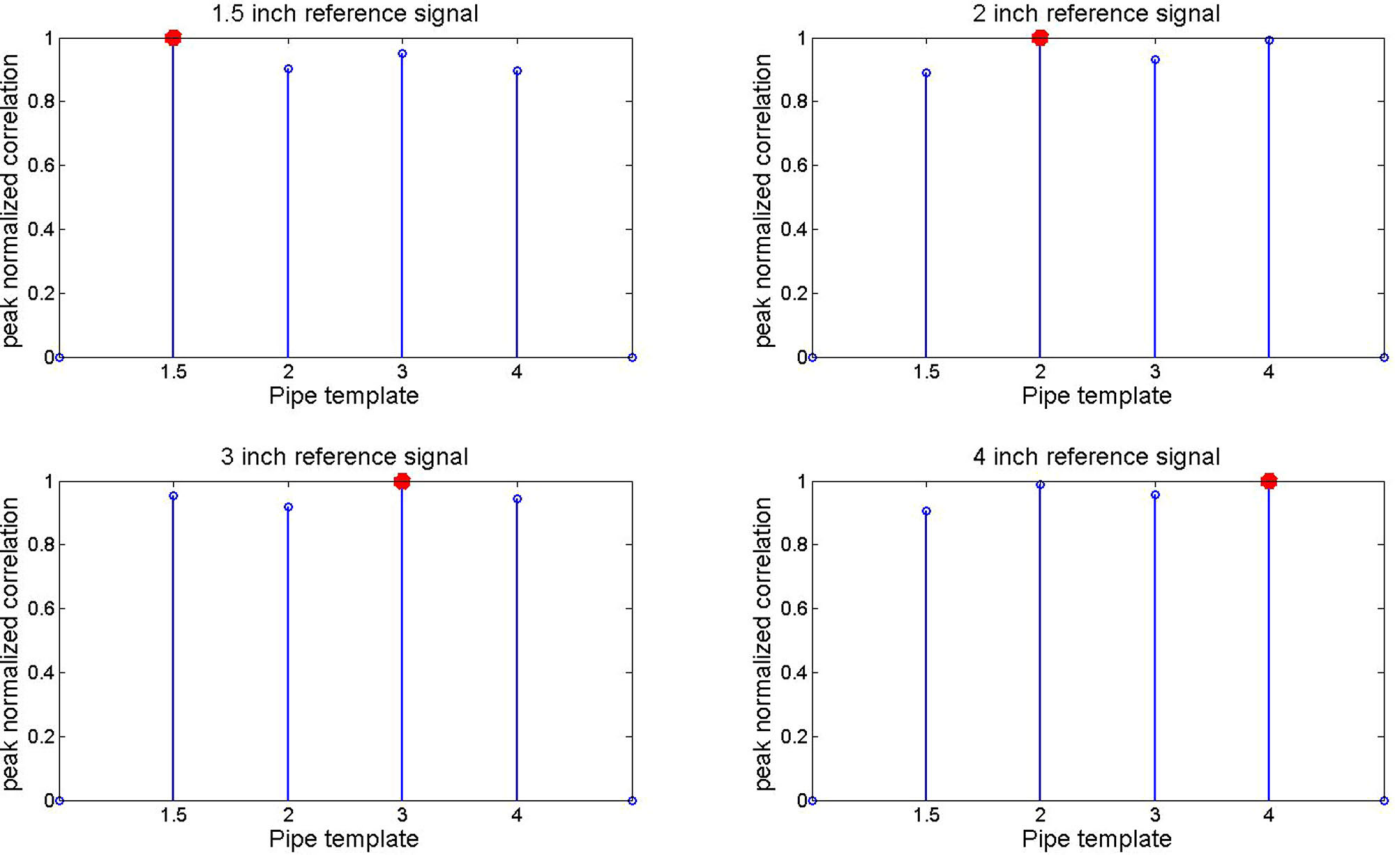
**Demodulated signal and reference signal peak correlations in mean frequency content of Eqn (5) are shown for all pipe diameters.** A red marker is used to denote the highest correlation in each template to the reference signal.

**Fig. 7.**
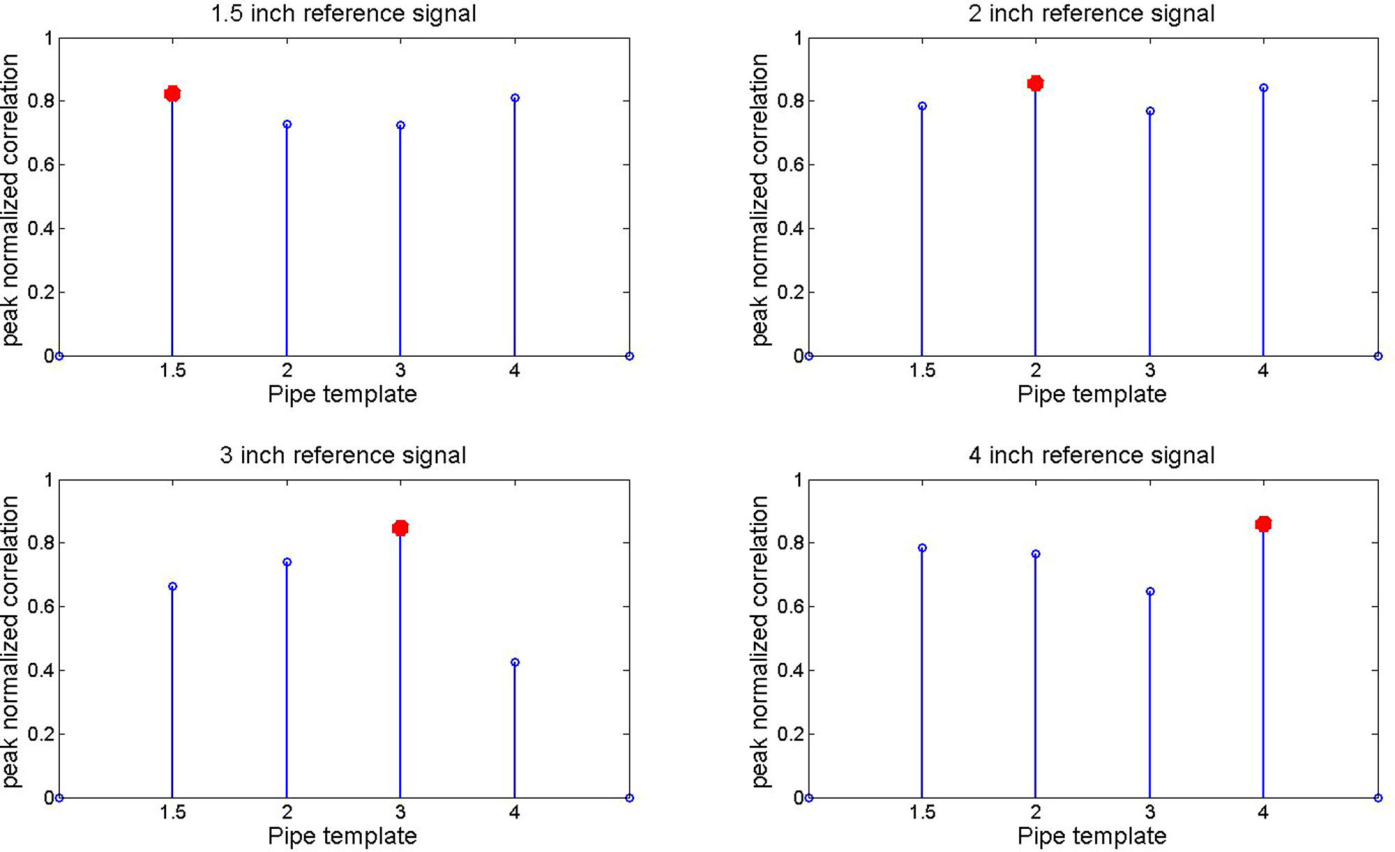
**Cyclic spectrum input signal and reference cyclic spectrum signal peak correlations in mean frequency content as described in Eqn (10) are shown for all pipe diameters.** A red marker is used to denote the highest correlation in each template to the reference signal.

Using the same variable notation where appropriate for continuity, the cross-spectral density and coherence models for cyclostationarity are applied. In particular, we consider the spectral density function as defined in Like (2007):
(6)
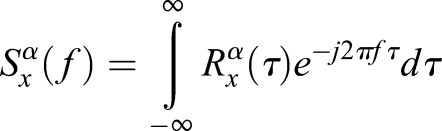
In this notation, the spectral content in *f* is differentiated from the cyclic content in α as defined in Napolitano (2012). This is important as we are now dealing in the cyclic-spectral perspective rather than time-spectral perspective, though the datasets remain the same. The implication is that we are now analyzing frequency content that presents with periodic behavior over time. When taking the autocorrelation of a signal, a real signal in time *x*(*t*) will be correlated with itself to produce
(7)
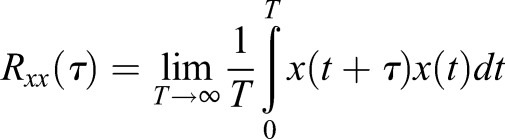
Given two input signals, it is possible to derive the cross-spectral coherence function for a given α band:
(8)
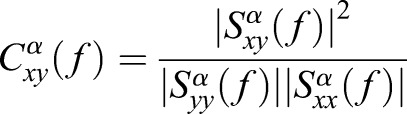
To achieve a template structure, a variation on (8) is applied in an effort to isolate cyclostationary elements associated with the air volume being actuated. In doing so, we derive cross-spectral density functions for both the modulated information signal *x* and the hypothesized demodulated information signal described *y* [see (3)]. The intent will be to derive the modulation signal estimated in (2). We then derive the spectral autocorrelation of a candidate modulated information signal *z*. The resulting coherent spectrum – not to be confused with the cross-spectral coherence in (8) – is used as a cross-correlating metric against known quantities of previously cross-spectrum deconvolved signals of known volume:
(9)
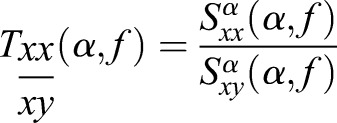
In this equation, *T* represents the coherent spectrum of a template signal *x* and its information deconvolved signal *y*. The intent of this exercise is to take the assumption that signals being shaped in the time domain are thus correlated in the frequency domain; deconvolving the input impulse (muscle input) will yield the desired characteristic impulse response modulation (bladder resonance). Ideally, the only elements left will be the residual modulation from the swim bladder as sought in (2). A template function for each specific volume to be tested must be created, forming a set of matched filters. When an input is applied to this model, it is possible to derive a coherent spectrum of signals which are expected to represent the residual carrier as sought in (2):
(10)
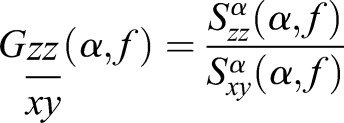
Once the decorrelated frequency content of *G* is derived about the unit regions of interest, it is averaged and cross-correlated with the same for each template volume *T* to derive match criteria in the same fashion as the preceding section, where the cyclic content is operated on in the place of time content.
(11)
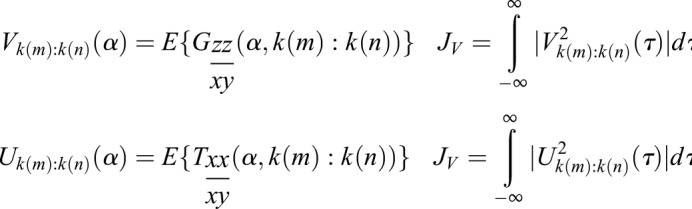
Operating on the cyclic frequencies, a normalized cross-correlation *Z*_2_ is derived for the average cyclic content in the spectral ROI of 150–200 Hz:
(12)
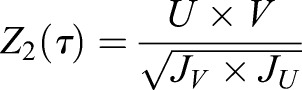
The resulting peak correlations of Z1 and Z2 are shown in Figs 6 and 7. In each dataset, the correct air volume can be ascertained from the peak correlation using either method. This suggests correlation in the modulation of each signal by the associated air volume.
